# A case of a primary pulmonary meningioma mimicking a metastasis from a papillary thyroid carcinoma due to a size reduction after radioactive iodine therapy

**DOI:** 10.1186/s40792-020-00823-y

**Published:** 2020-03-27

**Authors:** Ryo Fujikawa, Yoshifumi Arai, Yoshiro Otsuki, Toru Nakamura

**Affiliations:** 1 Department of General Thoracic Surgery, Seirei Hamamatsu General Hospital, 2-12-12, Sumiyoshi, Naka-ku, Hamamatsu, Shizuoka, 430-8558 Japan; 2 Department of Pathology, Seirei Hamamatsu General Hospital, Hamamatsu, Japan

**Keywords:** Primary pulmonary meningioma, Video-assisted thoracoscopic surgery, Metastatic lung tumor

## Abstract

**Background:**

Primary pulmonary meningiomas (PPMs) are a rare mostly benign disease presenting as a solitary pulmonary nodule and are hardly distinguishable from a metastatic tumor because of a lack of specific radiological features. We described a case of a PPM initially diagnosed as a metastatic lung tumor from thyroid cancer with a size reduction after radioactive iodine therapy.

**Case presentation:**

A 62-year-old woman who had undergone a total thyroidectomy for a papillary thyroid carcinoma 6 years prior presented with an enlarging pulmonary nodule. The nodule had decreased in size from 7.0 to 5.5 mm after adjuvant radioactive iodine therapy and enlarged to 8.7 mm over the next 5 years. Under a clinical diagnosis of a metastatic lung tumor, she underwent a thoracoscopic pulmonary wedge resection and was pathologically diagnosed with a PPM.

**Conclusion:**

A surgical resection is required for histological diagnoses of PPMs especially in patients with a history of a malignancy.

## Background

Primary pulmonary meningiomas (PPMs) are a rare and mostly benign disease and often present as a solitary pulmonary nodule [1, 2]. Because most PPMs grow in size without any specific radiological features, it is quite difficult to distinguish them from metastatic lung tumors in patients with a history of a malignancy [3–7]. We described a case of a PPM initially suspected as a metastasis from thyroid cancer due to a size reduction after radioactive iodine therapy.

## Case presentation

**Fig. 1:**
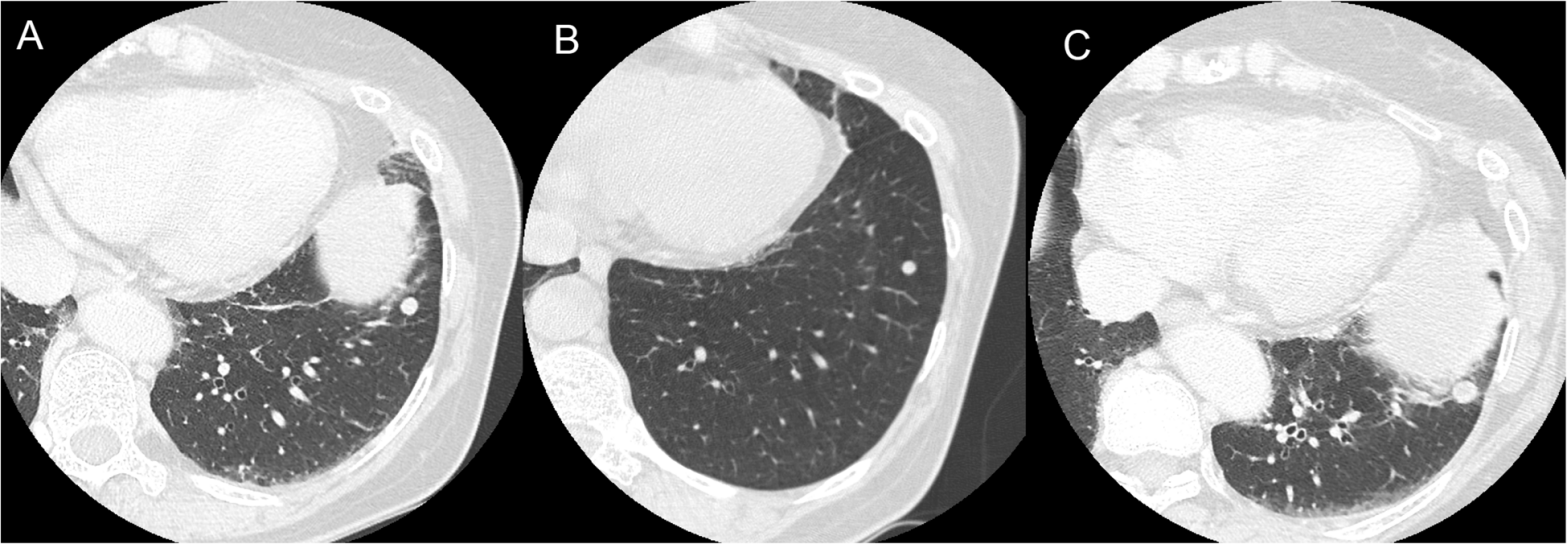
Computed tomography showing a peripheral well-circumscribed pulmonary nodule in the left lower lobe. **a** The size was 7.0 × 6.6 mm before the thyroidectomy. **b** It decreased in size to 5.5 × 5.0 mm after the adjuvant radioactive iodine therapy. **c** It grew to 8.7 × 8.0 mm over 5 years

**Fig. 2:**
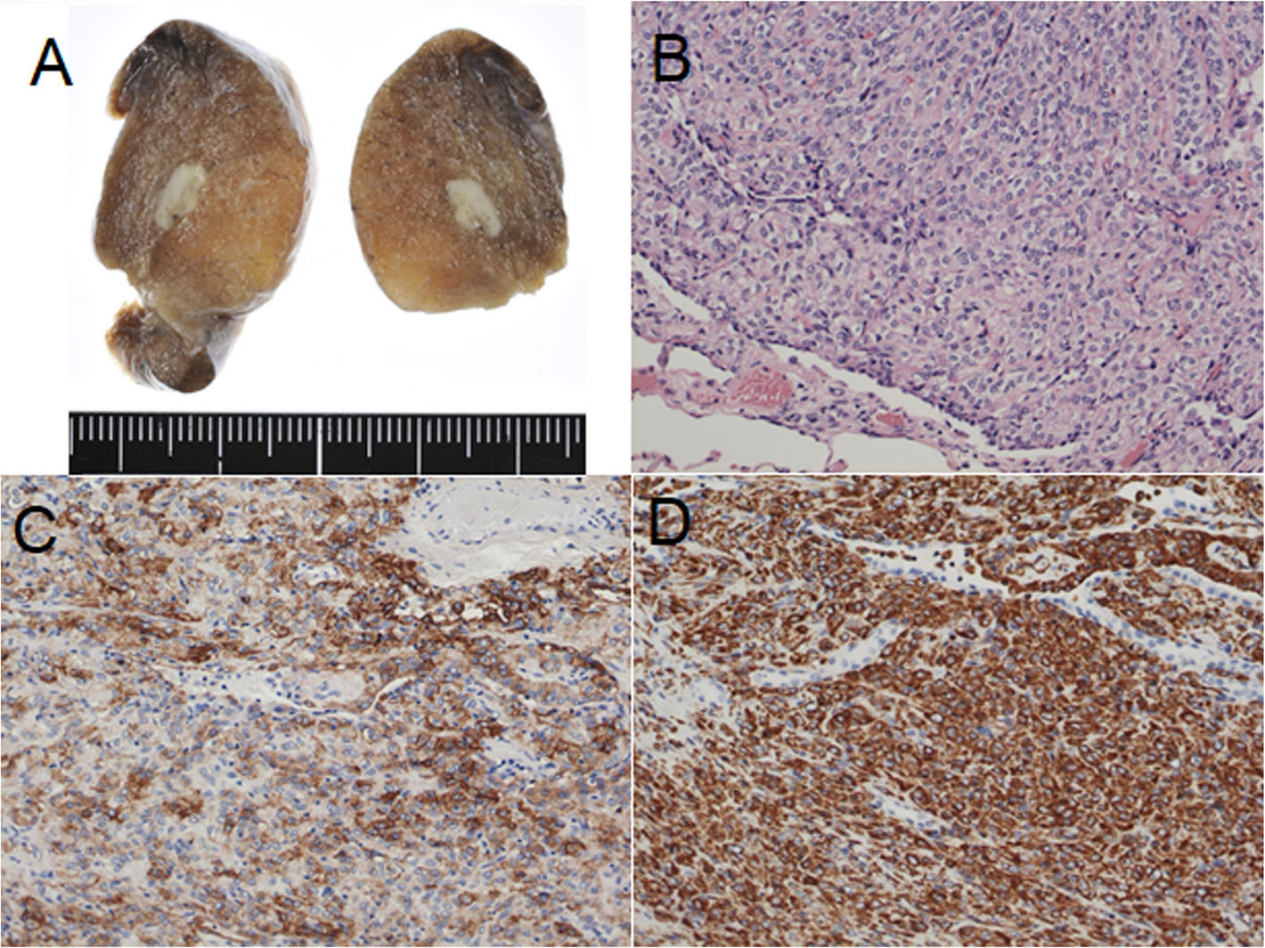
**a** A gross finding of the surgical specimen revealed a well-circumscribed white nodule measuring 8 mm. **b** The neoplastic cells with oval nuclei are arranged in sheets (hematoxylin and eosin stain). **c** The neoplastic cells were positive for epithelial membrane antigen, **d** and vimentin

A 62-year-old asymptomatic woman presented with an enlarging pulmonary nodule. She had undergone a total thyroidectomy and neck lymph node dissection for a stage IVC (T4aN0M1) papillary thyroid carcinoma 6 years prior. Computed tomography before the thyroidectomy had revealed a peripheral well-circumscribed pulmonary nodule in the left lower lobe which was suspected as a metastasis (Fig. 1a). The nodule had decreased in size from 7.0 to 5.5 mm after adjuvant radioactive iodine therapy (Fig. 1b) and enlarged to 8.7 mm over the next 5 years (Fig. 1c) (Each figure showed the maximum diameter of the nodule. The difference of slice levels might be caused by a discordance in respiratory conditions.). There were no other pulmonary nor mediastinal abnormalities. Both transbronchial and percutaneous biopsy seemed nondiagnostic due to the small size and peripheral location of the nodule. She underwent a thoracoscopic pulmonary wedge resection with an uneventful recovery. A gross examination showed a well-circumscribed white nodule measuring 8 mm (Fig. 2a). Microscopic examination revealed that there were neoplastic cells with oval nuclei arranged in sheets without mitotic figures nor atypia (Fig. 2b). Whorled clusters or psammoma bodies were absent. Immunohistochemical stains were positive for epithelial membrane antigen, vimentin, and progesterone receptors (Fig. 2c, d). The negativity for cytokeratin 7, cytokeratin 20, thyroglobulin, paired box gene 8, chromogranin A, and synaptophysin made a metastasis from thyroid cancer, primary lung cancer, and a neurogenic tumor less likely. The pathological diagnosis was a meningioma without malignant characteristics. Because postoperative cerebral and spinal magnetic resonance imaging showed no sign of a meningioma, we finally diagnosed the nodule as a PPM. She currently has been disease free by the 20-month follow-up evaluation.

### Comment

PPMs are rare and only 46 cases (including our case) have been reported in English literature since the first case was described in 1981 [7]. Most of those cases were asymptomatic, grew slowly, and had an excellent prognosis with an unknown etiology. Because intracranial or spinal meningiomas can metastasize to the lungs, which is the most frequent target [8, 9], radiological studies of the central nervous system are essential to identify PPMs.

Histologic features of PPMs are similar to intracranial and intraspinal meningiomas. Cells with oval nuclei are arranged in sheets, whorls, or onion peel-like structures. Psammoma bodies are often present. Immunostaining shows positivity for epithelial membrane antigen and vimentin. In our case, the negative findings of the immunostaining excluded differential diagnoses, and both of the histological features as cells arranged in sheets and the positivity for epithelial membrane antigen and vimentin led us to diagnosis of meningioma.

Typical radiological findings of PPMs show a solitary, well-circumscribed, and non-calcified nodule with sizes ranging from 0.4 to 6 cm [2]. They can mimic metastatic lung tumors in patients with a history of a malignancy because of the lack of specific radiological findings. Positron emission tomography is not useful to distinguish these two pathologies because of an increased F-18-fluorodeoxyglucose uptake with PPMs [2, 10–12]. A cytologic diagnosis such as by transthoracic needle biopsy may also be difficult due to its rarity [2]. Therefore, a surgical resection is required to diagnose and also could be therapeutic. Because solitary pulmonary nodules in patients with a history of a malignancy have a possibility of a primary lung cancer beside a metastatic lung tumor, we consider that a thoracoscopic biopsy is a feasible option [13–15].

In our case, we initially diagnosed the nodule as a metastatic lung tumor because of the size reduction after radioactive iodine therapy despite the rarity of solitary pulmonary metastases from thyroid cancer [16]. We considered there was no correlation between the radioactive iodine therapy and the PPM without any established evidence in a literature. Our case provided an important clinical issue that a PPM could reveal a spontaneous transient regression by unknown etiology, which revealed seemingly a radiological response to the radioactive iodine therapy. Regardless of a lung metastasis, radioactive iodine therapy was applied due to its local invasion in our case, but unnecessary chemotherapies could be administered for a PPM suspected as a metastasis only by the radiological diagnosis [4, 5]. A correct diagnosis by a surgical resection would prevent an overdiagnosis and overtreatment even if a solitary pulmonary metastasis is clinically suspected.

## Conclusion

A temporary size reduction after radioactive iodine therapy led us to a misdiagnosis of a pulmonary metastasis in our case. A surgical resection is necessary to avoid an overdiagnosis even if a solitary pulmonary metastasis is suspected.

## Data Availability

Not applicable.

## Abbreviations

PPM: Primary pulmonary meningioma
